# Breastfeeding Practices and Food Consumption of Socially Vulnerable Children

**DOI:** 10.3390/foods14010138

**Published:** 2025-01-06

**Authors:** Natália A. Oliveira, Nathalia Pizato, Érika S. O. Patriota, Ariene S. do Carmo, Gabriela Buccini, Vivian S. S. Gonçalves

**Affiliations:** 1 Graduate Program in Public Health, Faculty of Health Sciences, University of Brasília, Brasília 70910-900, DF, Brazil; natyunb.2010@gmail.com (N.A.O.); erikapatriota.nut@gmail.com (É.S.O.P.); vivian.goncalves@unb.br (V.S.S.G.); 2 Graduate Program in Human Nutrition, Faculty of Health Sciences, University of Brasília, Brasília 70910-900, DF, Brazil; arienecarmo@gmail.com; 3 Department of Social and Behavioral Health, School of Public Health, University of Nevada, Las Vegas, NV 89154, USA; gabriela.buccini@unlv.edu

**Keywords:** food consumption, child development, home visits, breastfeeding, health promotion

## Abstract

Promoting child well-being and development requires a multidimensional approach, including the right to adequate food practices. Socially vulnerable children are more exposed to adverse experiences, such as inadequate food consumption due to poverty. In this context, home-visiting programs are an important strategy for nutritional and health care education to provide relevant guidelines. This study describes breastfeeding and food consumption of children aged 0 to 24 months assisted by the Happy Child Program (Programa Criança Feliz—PCF) and aimed to investigate their association with socioeconomic factors and adherence to the program. This is an observational study, with a cross-sectional design, carried out with children assisted by the PCF in the Federal District, Brazil. Multivariate analysis was performed to identify sociodemographic and income factors, and household visits characteristics associated with breastfeeding, dietary diversity, and the consumption of ultra-processed food. A total of 301 children were assessed, 51.16% of whom were female. In 58.99% of households, the reference person was the mother; 86.20% were unemployed, and 27.08% had a low education degree. About 62.65% of beneficiaries lived on up to USD 200.00 per month and the majority faced food insecurity. The child’s age, and the mother’s current work situation were independently associated with the current breastfeeding situation, especially higher among women who did not work (*p* = 0.015). The minimum dietary diversity among children over 6 months old was 62.21% and presented a positive association with adherence to the program (*p* = 0.005). On the other hand, the consumption of ultra-processed foods was 77.21% and was associated with a longer follow-up time within the program (*p* = 0.047). The associations observed revealed the need to integrate family food choices and nutritional education into public policies for early childhood.

## 1. Introduction

Promoting child development (CD) requires a multidimensional approach intrinsically linked to the interdependence of human rights, including the human right to adequate food (DHAA) [1,2]. This comprehensive and integrated care is particularly relevant given the evidence that the first thousand days of life, from pregnancy until 24 months of age, represent a critical window of opportunity for promoting long-term health [3,4,5,6].

Despite progress in guaranteeing DHAA in Brazil, several challenges remain regarding adequate infant nutrition. At least one in three children under five worldwide is affected by one or more forms of malnutrition—undernutrition, overweight, and micronutrient deficiencies [7]. In Brazil, only four in ten children are exclusively breastfed for up to six months, and 80% of children between 6 and 23 months consume ultra-processed foods (UPFs) [8]. These dietary patterns are directly related to the prevalence of overweight (7%) and obesity (3%) among children under five [8].

In this context, home visitation programs have emerged as a relevant policy directive for promoting CD, gaining prominence in public health and child protection agendas, as well the Sustainable Development Goals (SDG) objectives [9,10]. These programs facilitate access to preventive and curative health services and promote parental knowledge of social protection services, food security, and opportunities for early intervention [11].

In the global context, home-visiting programs have been successfully implemented in various countries, such as Healthy Families New York (HFNY) in the United States and the Eduque a su Hijo program in Cuba. These initiatives have demonstrated positive impacts on maternal and child health, strengthening parent–child bonding, and improving nutritional outcomes [12,13]. In low-income countries such as South Africa, similar programs have shown great potential to reduce malnutrition and promote breastfeeding, particularly in vulnerable communities, by combining home visits with nutritional education and social assistance initiatives [14].

The Happy Child Program (Programa Criança Feliz—PCF) is a Brazilian initiative to strengthen parental skills and promote comprehensive child care through interdisciplinary actions. This program can potentially be an effective tool for promoting early childhood care in the areas where it is implemented [15].

Recent studies conducted in Brazil have demonstrated the limited impact of the PCF on the overall CD outcomes [16,17], highlighting the importance of more comprehensive investigations into factors that could synergistically influence CD, such as those related to adequate nutrition. Therefore, this study sought to describe breastfeeding (BF), dietary diversity, and the consumption of UPF indicators among children aged 0 to 24 months assisted by the PCF in the Federal District, investigating their association with socioeconomic indicators and program adherence.

## 2. Materials and Methods

This study followed the Strengthening the Reporting of Observational Studies in Epidemiology (STROBE) guidelines [18]. The STROBE checklist can be found in the Supplementary Material.

### 2.1. Study Design

This is a cross-sectional observational study conducted between May and July 2022 based on the baseline of the cohort study titled “Evaluation Project of the Implementation of the Happy Child Program in the Federal District (PIPA-DF)”.

### 2.2. Context and Participants

This study included socially vulnerable children from the Federal District (FD) who participated in the PCF. To be eligible for the PCF, families had to be registered in the Unified Registry (CadÚnico) and receive support from a local Social Assistance Reference Center (CRAS) [19]. The PCF was characterized by regular visits conducted by trained professionals to pregnant women and families with children up to 36 months old.

#### 2.2.1. Sample Size

The sample size calculation considered the proportion of 11.8% of children under 36 months with a Z-score ≥ 1, obtained from the “Primeira Infância para Adultos Saudáveis (PIPAS)” study developed in 2018 [20]. The following were also considered: a sampling error (d) of 5%, Z equal to 1.96 corresponding to a 95% confidence level, and a design effect (deff) of 1.5. Thus, the estimated sample size for the study was 300 children. The algebraic expressions used for the calculation are presented below [21].

n = (p∙(1 − p))/(d/z)^2∙deff → d = √((p∙(1 − p)∙z^2∙deff)/n)

#### 2.2.2. Eligibility Criteria

All families surveyed with children up to 24 months of age were considered eligible for the evaluations. The youngest child was selected in families with more than one child between 0 and 24 months. The inclusion criteria were being eligible for the program throughout the entire research period. Children from families whose caregivers could not respond to the questionnaires due to any cognitive deficit or who were beneficiaries of the Continuous Cash Benefit (BPC) were excluded.

### 2.3. Variables and Measurement

The data collection instrument employed was a structured questionnaire adapted from the PIPAS study [20]. Questions adapted from the “nutrition” and “protection and safety” components of the QAD-PIPAS were utilized to define the outcome and exposure variables. To ensure data collection accuracy and consistency, the interviewers underwent comprehensive training, which included an introduction to the instrument, study protocols, and field collection procedures. Interviews were conducted in person with the children’s caregivers at their households, with support from PCF visitors and supervisors. The questionnaire was administered digitally using the KoboCollect^®^ application facilitated by smartphones.

#### 2.3.1. Breastfeeding and Food Consumption Indicators (Outcome Variables)

Breastfeeding practices among children aged 0 to 24 months were measured through two questions: (1) Is the child still being breastfed? (yes, no, never breastfed, or do not know); and (2) How long did the child breastfeed exclusively, without water, tea, or other liquids? (in months).

The analysis was based on the Ministry of Health’s (MH) guidelines [22], which are grounded in indicators for evaluating infant and child feeding practices from the World Health Organization (WHO) [23], as well as the PIPAS study, with adaptations [20]:

(1) Current breastfeeding status: the proportion of children aged 0 to 24 months who were receiving breast milk on the day of the evaluation, regardless of exclusivity. This variable was selected for the Poisson regression model;

(2) Exclusive breastfeeding (EBF): the proportion of children up to 6 months who were breastfed without receiving water, tea, or other liquids, or children who were exclusively breastfed until 6 months of age;

(3) Continued breastfeeding (CBF): the proportion of children aged 6 to 24 months who were receiving breast milk on the day of the evaluation.

Dietary diversity and the consumption of UPFs were assessed through questions with three response options (yes, no, or do not know) regarding food consumption on the day prior to the interview. Healthy food consumption markers evaluate the consumption of beans, rice or potatoes, fruits, vegetables, milks, red meat, or eggs and unhealthy food consumption markers are regular consumption of sweetened beverages and confectionery and excessive salt. For the analyses, food consumption markers were used as defined based on the MH’s Food Consumption Markers proposal [22], with adaptations:

(1) Minimum dietary diversity: the proportion of children aged 6 to 24 months who consumed five food groups on the day prior to the interview, divided by the total number of children in the same age group. The evaluated food groups were (1) grains, roots, and tubers; (2) legumes; (3) breast milk, non-breast milk, and dairy products; (4) meats and eggs; and (5) fruits, vegetables, and greens;

(2) Consumption of ultra-processed foods (UPFs): the proportion of children aged 6 to 24 months who consumed at least one UPF the day before the interview, divided by the total number of children in the same age group. The evaluated UPFs were (1) soda or boxed juice; (2) savory/sweet cookies; (3) packaged snacks; (4) candy/lollipops/chocolate/sweets.

#### 2.3.2. Demographic, Socioeconomic, and PCF Adherence Indicators (Exposure Variables)

The demographic and socioeconomic variables, along with their response categories, were: the child’s date of birth (used to calculate age in months); the child’s sex (female and male); the child’s race/color (white, black, brown, yellow, indigenous, or do not know); the caregiver’s age (in years); the reference person/head of household (father, mother, or other); maternal education (no education, incomplete primary education, complete primary I/incomplete primary II, complete primary II/incomplete secondary, complete secondary/incomplete higher education, complete higher education, or do not know/do not remember); maternal occupation (employed, unemployed, retired, or on paid maternity leave); and household income (under to USD 100.00, between USD 100.00 and USD 200.00, between USD 200.00 and USD 400.00, >USD 400.00, or do not know/did not answer).

For analysis purposes, the child’s race/color was categorized as white and non-white (black, brown, and Indigenous). Given the low representation of children of Asian descent (<1%), indicator estimates for these subgroups were not presented. The child’s age was stratified to account for dietary diversity in each age group [24]. The caregiver’s age was categorized for analysis, and maternal education was classified into low, medium, and high education based on the first three levels of education defined by the Institute of Applied Economic Research (IPEA) [25].

Adherence to the PCF was measured using five questions to assess the program’s “doses”. Positive or higher response categories were used as the reference: (1) “How long has the child been receiving PCF visits?” (in months, categorized into up to 8 months and 9 months or more for analysis); (2) “How many PCF visits did the child receive in the last month?” (4 visits—1 per week—or <4 visits); (3) “What is the duration of the visits the child receives from PCF?” (60 min or <60 min); (4) “After the visit, does the family carry out the activities proposed by the visitor with the child?” (yes or no); and (5) “How much do you trust (believe in) the guidance you receive from PCF?” (I fully trust and follow; I partially trust; indifferent; and I do not trust and do not follow). For the analysis, the categories “I partially trust, indifferent, and I do not trust and do not follow” were combined into a single category of partial or no trust.

Food insecurity (FI) in households was assessed using the Brazilian Food Insecurity Scale (EBIA). The possible responses to this scale are “yes” and “no”, with the score of affirmative responses determining the classification of households into food security (0), mild insecurity (1 to 5), moderate insecurity (6 to 9), and severe insecurity (10 to 14) [26].

### 2.4. Data Collection Bias

The data quality control stage was conducted rigorously and in real-time to identify any systematic errors arising from data collection. The data were checked daily to prevent measurement failures or incompleteness, and when necessary, additional data collection was carried out.

### 2.5. Statistical Methods

Descriptive data analysis was performed, calculating prevalence with a 95% Confidence Interval (CI) for categorical variables. The mean age of the children and mothers was presented with their respective standard deviations (SD). Poisson Regression with robust variance was used to calculate prevalence ratios (PR) in the association analyses between the exposure variables (demographic, socioeconomic, and PCF adherence) and outcomes (current breastfeeding status, minimum dietary diversity, and UPF consumption). Associations with a *p*-value less than 0.20 in the univariate analysis were selected for inclusion in the multivariate analysis.

The backward method was used in the final model, where variables with lower significance (higher *p*-value) were removed individually. This procedure was repeated until all variables in the model had statistical significance (*p* < 0.05). All statistical analyses were conducted using STATA^®^ software version 16, taking sample weights into account.

### 2.6. Ethical Considerations

Since this study involved human subjects, PIPA-DF was submitted for review by the Research Ethics Committee of the Faculty of Health Sciences at the University of Brasília (CAAE: 32390620.0.0000.0030), and the children’s guardians signed the Free and Informed Consent Form (ICF) prior to data collection.

The study adhered to the ethical principles outlined in Resolution n° 738/2024 of the National Health Council (NHC) [27], minimizing risks, maximizing benefits, and ensuring the privacy and confidentiality of participant data.

## 3. Results

A total of 301 children were assessed, with an average age of 14.54 months (SD = 5.92). Of these, 51.16% were female, and 69.25% were non-white (Black, Brown, and Indigenous). In more than half of the households (58.99%), the reference person (head of the family) was the mother, with an average age of 29.31 (SD = 8.75) years. Most mothers (86.20%) were unemployed, and 27.08% had low education levels. Regarding family income, 62.65% of the beneficiaries lived on up to USD 200.00 per month. The application of the EBIA showed that 83.87% of the households experienced some degree of IA, with 49.33% in mild IA, 23.11% in moderate IA, and 11.43% in severe IA (Table 1).

Table 1 also presents data on the beneficiaries’ adherence to the PCF. More than half had been followed for less than 8 months and receiving the recommended number of visits (1 per week) [28]. Almost all beneficiaries reported engaging in the activities proposed by the visitor and trusting and following the provided guidance.

Regarding breastfeeding practices, 37.33% of mothers reported not breastfeeding at the time of the interview (Table 2). Table 2 also presents the status of exclusive and continued breastfeeding among the children benefiting from the program.

Minimum dietary diversity and consumption of ultra-processed foods (Table 2) were also assessed among children older than 6 months, with 62.21% exhibiting minimum dietary diversity. Regarding the consumption of ultra-processed foods, it was found that 77.21% had consumed at least one type of these foods on the previous day.

The crude analysis revealed associations between demographic, socioeconomic, and PCF adherence variables with children’s dietary indicators. The current status of breastfeeding was independently associated with the child’s age and the mother’s current employment status, with a higher prevalence among non-working women. In addition, the prevalence ratio of breastfeeding was lower among children from families with higher income levels (Table 3).

Minimum dietary diversity was higher among children receiving four visits in the past month and non-white children (Table 4). Conversely, the consumption of ultra-processed foods was higher among those with longer follow-ups by the PCF and lower among male children (Table 5). In the multivariate analysis, these associations remained significant (*p* < 0.05).

## 4. Discussion

This study investigated BF practices and dietary diversity and the consumption of UPFs among children aged 0 to 24 months in situations of social vulnerability, focusing on the association with adherence to a child development promotion program and other social, economic, and demographic factors. Most participants had low incomes and lived in situations of food insecurity; nearly all participated in the activities and trusted the guidance provided by the visitors. Our results show that program adherence is associated with greater dietary diversity. However, the same benefit was not observed for BF practices or the consumption of UPFs.

Despite the World Health Organization (WHO) and the MH recommending EBF until six months of age [24,29] and, after that, the inclusion of appropriate foods alongside breast milk [30], the EBF rate among beneficiaries up to six months of age reached only 53.13%, while CBF reached 60.23% among those aged 6 to 24 months. These rates were similar to those of children followed in Primary Health Care in 2023, where the prevalence of EBF was 55% and CBF was 61% [31], and higher than the prevalences observed in the National Study of Food and Child Nutrition (ENANI-2019), which were 45.8% for EBF and 43.6% for MCF among children aged 12 to 23 months in Brazil, and 46.5% for EBF and 43.9% for MCF in the Central-West region [11]. The prevalence of BF was higher among unemployed mothers, corroborating studies that identify employment as a primary factor for early weaning [32,33,34]. Oliveira et al. (2022) [32] found that 80.5% of mothers who practiced exclusive breastfeeding were not employed, and 72% of mothers who practiced predominant breastfeeding were also not employed. To ensure the continuation of breastfeeding after returning to work, structural barriers must be overcome, and it is essential that women are prepared during prenatal care to continue breastfeeding [35].

Additionally, it is crucial to consider obstacles such as job security, maternal education, and local government support. According to Santos et al. (2021) [36], women in the informal sector without access to maternity leave have a high probability of discontinuing breastfeeding. In other cases, when women assume the role of head of household, they are often required to work outside the home, which also leads to the interruption of breastfeeding [37]. Therefore, comprehensive policies that promote breastfeeding-friendly work environments, public childcare facilities, and health promotion strategies are essential for sustaining breastfeeding [32]. Women should also be informed of their rights to reduced working hours and breaks for milk expression or breastfeeding [38], which could be reinforced by the PCF through professional, supportive approaches, as outlined in the program’s guidelines [39].

It is important to highlight that CBF, up to two years or beyond, provides numerous health benefits for children under five, protects against infectious diseases, reduces the risk of overweight and obesity, and contributes to cognitive development [40,41]. However, the lack of program support may have limited effective interventions, influencing the BF outcomes. These results are supported by the qualitative evaluation study of the PCF conducted by Dos Santos et al. (2023) [10], in which early weaning was frequently reported by families and attributed to a variety of factors, including lack of support and information about breastfeeding.

Regarding minimum dietary diversity, most beneficiaries adhered to the Brazilian Ministry of Health’s recommendations for children under two [24]. These findings are similar to those of Lacerda et al. (2023) [42], where the prevalence of minimum dietary diversity was 63.4% among children aged 6 to 23 months, and they exceed the data from the ENANI-2019 study [11] and Spaniol et al. (2020) [43], which reported that 55.6% and 44.6% of children aged 12 to 24 months achieved minimum dietary diversity, respectively.

Minimum dietary diversity was associated with the number of PCF visits in the previous month, demonstrating that those who were followed according to the recommendation (one visit per week) [28] were more likely to follow the guidelines for promoting adequate and healthy eating. Additionally, minimum dietary diversity was more prevalent among non-white beneficiaries. This association corroborates a study based on the Household Budget Survey, which shows that in more vulnerable populations of the country, such as among Black and Brown individuals, there is a higher consumption of in natura and minimally processed foods [44]. However, it is important to highlight that within this population, there has been a faster growth rate of ultra-processed foods over the years [45].

Home visits are aimed at situations where rights and bonds have already been violated and those that are preventive, protective, and proactive in ensuring rights [46]. In this way, when visitors identify socially vulnerable families, they may facilitate access to adequate and healthy food through nutritional guidance and connect families with food and nutritional security resources in their communities [14], which could explain the observed dietary diversity.

In contrast, the data on UPF consumption is concerning, as 77.21% of the children consumed some type of these foods the day before the interview, and it was also associated with longer participation in the PCF (*p* = 0.047). Evidence has shown that UPFs are being introduced increasingly early in the feeding of children under two, both in Brazil and in other countries [47,48]. In 2019, the prevalence of UPF consumption among children aged 6 to 23 months was 80.5% [11], and in 2021, the prevalence among children under two years remained high at 72% [49]. Similar trends are also observed globally, with rising prevalence of UPF consumption during early childhood. According to data from the National Health and Nutrition Examination Survey (NHANES) in the United States, the estimated percentage of total energy intake from UPF consumption among children increased from 61.4% in 1999–2000 to 67.0% in 2017–2018 [50]. In Argentina, the consumption of processed and ultra-processed foods among children was lower compared to Brazil, where the prevalence reached 36% [51]. The harmful effects associated with UPF consumption in childhood are numerous, including impacts on development [52] and growth [53,54], respiratory problems [55], childhood overweight and obesity [56], and other consequences of poor nutrition, such as anemia and other consequences of poor nutrition [57]. Thus, the results suggest that although the home visits promote healthy and diverse foods, they may also unintentionally influence UPF consumption, which could be related to the lack of clear guidance from program visitors regarding the harmful effects of these foods. In various regions around the world, children have adopted predominantly unhealthy eating habits, marked by the frequent consumption of ultra-processed foods at the expense of a healthy and diverse diet. This practice, combined with other risk factors, is associated with an increase in childhood obesity, a high prevalence of sedentary behavior, and a greater risk of developing non-communicable chronic diseases, such as cardiovascular issues and dental cavities [44,58,59,60].

The scenario in which most children presented both dietary diversity and high consumption of UPFs concurrently can be further explained by the food choices of socially vulnerable groups. These choices are often based on foods that provide a greater sense of satiety, such as rice and beans, and a preference for low-cost foods, which are frequently rich in empty calories, such as UPFs, fast food, and products high in sugar and fat. The results can also be justified by a relatively high level of awareness about the need for adequate nutrition, as promoted by social assistance programs, but are related to the significant difficulty families face in identifying what is healthy and interpreting food labels [61].

However, addressing UPF consumption among children requires an intersectoral approach, as it is linked to a complex network of factors beyond the mere lack of information [62]. Public policies should focus on creating incentives to make healthy foods more accessible, including subsidies for producing and distributing these foods and financial support programs for vulnerable families [63,64,65].

Moreover, it is essential to implement stricter regulations on the advertising of UPFs to children [66] and promote food and nutrition education programs that help families overcome the lack of cooking skills, time constraints, and foster a healthy family environment [67]. These findings reinforce the idea that earlier interventions are needed to promote healthy eating habits, including nutrition education initiatives in policies aimed at the comprehensive development of early childhood, a phase that is most conducive to health promotion and disease prevention [68,69]. Therefore, home visitation programs can strengthen the promotion of adequate nutrition, contributing to better child development, since the visitors are well-informed about these guidelines. The coordination of these intersectoral strategies, combined with effective actions to combat malnutrition and promote breastfeeding, plays a crucial role in improving child nutrition and mitigating negative impacts in this age group [70].

This study presents an innovative aspect by addressing issues related to BF, food consumption, and their association with socioeconomic factors and adherence to a child development promotion program. Although the study’s cross-sectional nature does not allow for causal inferences about the observed associations, it serves as an important starting point for future longitudinal studies that can conduct more robust causal analyses. One limitation is that the analysis of food consumption is considered to be only one day, which may not fully reflect the children’s eating habits. Another possible limitation is the discomfort in discussing food access or income. To minimize these impacts, efforts were made to clarify the research question and ensure voluntary participation. It is also worth mentioning that the results obtained are specific to PCF participants, which limits generalization to the FD or to the Brazilian population as a whole.

Nevertheless, the results come from a representative sample of socially vulnerable children and reveal a previously unmonitored situation-BF practices and eating habits among these beneficiaries. Despite advances in child development policies, the associations highlight the need to integrate the promotion of adequate nutrition into public policies. In this regard, it is recommended that program visitors be trained on the topic, enabling them to promote nutrition education actions and advise families on healthy eating in the early years of life.

## 5. Conclusions

The child’s age and maternal employment status were significantly associated with the current breastfeeding situation, highlighting the importance of these factors in promoting breastfeeding. Furthermore, the observed minimum dietary diversity and the consumption of UPFs among children over six months indicate the need for specific food and nutrition education specific interventions to ensure adequate nutrition in this age group. In this context, it is crucial to emphasize the need for more comprehensive education for mothers and guardians, particularly focusing on healthy eating practices and the importance of breastfeeding. Expanding support options for mothers, such as offering accessible resources, counseling and support, would further strengthen these efforts and promote better nutritional outcomes for children.

## Figures and Tables

**Table 1:** Sociodemographic, economic, and adherence characteristics of the Happy Child Program beneficiaries. Federal District, Brazil, 2022.

Variables	%	95% CI
Child’s age		
Up to 6 months	10.83	7.74–14.96
6 to 12 months	23.72	19.16–28.97
13 to 18 months	35.64	30.28–41.38
19 months or more	29.81	24.75–35.41
Child’s gender		
Female	51.16	45.36–56.93
Male	48.84	43.06–54.64
Child’s race/color		
White	30.75	25.65–36.37
Non-white ^1^	69.25	63.63–74.35
Family reference person		
Father	28.69	23.67–34.30
Mother	58.99	53.14–64.60
Other	12.32	8.93–16.74
Age of the responsible person		
Until 19 years	6.00	3.76–9.42
20 to 29 years	52.30	46.49–58.05
30 to 39 years	29.56	24.52–35.15
≥40 years	12.14	8.83–16.45
Mother’s education		
Low level of education ^2^	27.08	22.18–32.61
Moderate level of education ^3^	22.92	18.41–28.15
High level of education ^4^	50.0	44.19–55.81
Mother’s current employment status		
Employed	13.80	10.31–18.23
Unemployed	86.20	81.77–89.69
Household income (USD)		
Under to 100.00	26.95	22.02–32.54
Between 100.00 and 200.00	35.70	30.31–41.48
Between 200.00 and 400.00	32.54	27.16–38.42
>400.00	4.81	2.76–8.23
Food (In)Security		
SAN	16.13	12.25–20.93
Slight FI	49.33	43.55–55.12
Moderate FI	23.11	18.52–28.45
Grave FI	11.43	8.20–15.69
PCF attendance time		
Up to 8 months	57.10	51.25–62.76
9 months or more	42.90	37.24–48.75
Last month’s visit frequency		
Less than 4 visits a month	9.93	6.98–13.93
4 visits (once a week)	90.07	86.06–93.01
Visit duration		
Less than 60 min	85.57	80.86–89.28
60 min	14.43	10.71–19.14
Practice activities proposed by PCF visitor		
No	5.27	3.27–8.39
Yes	94.73	91.61–96.73
Trust in received orientations		
Do not trust or trust partially	4.63	2.73–7.75
Trust completely and practice them	95.37	92.25–97.27

^1^ Non-white: black, brown, and indigenous; ^2^ low education: illiterate, incomplete/completed primary education level I, and incomplete lower secondary education; ^3^ medium education: completed lower secondary education or incomplete upper secondary education; ^4^ high education: completed upper secondary education and incomplete/completed higher education; 95% CI = 95% confidence interval; PCF = Happy Child Program (Programa Criança Feliz).

**Table 2:** Prevalence of breastfeeding, minimum dietary diversity, and consumption of ultra-processed foods among socially vulnerable children assisted by the Happy Child Program. Federal District, Brazil, 2022.

Variables	%	95% CI
Breastfeeding (actual status)		
Yes	62.67	56.88–68.12
No	37.33	31.89–43.12
Exclusive breastfeeding (until 6 months)		
Yes	53.13	32.48–72.77
No	46.87	27.23–67.52
Sustained breastfeeding (6 to 24 months)		
Yes	60.23	54.14–66.02
No	39.77	33.98–45.85
Minimal food diversity (6 to 24 months)		
Absence of food diversity	37.79	31.96–43.98
With food diversity	62.21	56.02–68.03
Ultra-processed food consumption (6 to 24 months)		
Did not take any	22.79	18.12–28.26
Had consumed at least one	77.21	71.74–81.88

95% CI = 95% confidence interval.

**Table 3:** Demographic, socioeconomic, and Happy Child Program adherence characteristics associated with breastfeeding. Federal District, Brazil, 2022.

Explanatory Variables	Breastfeeding *					
	Crude Analysis			Multivariate Analysis		
	PR	95% CI	*p*-Value	PR	95% CI	*p*-Value
Child’s age						
Up to 6 months	Ref.			Ref.		
6 to 12 months	0.08	0.69–1.05	0.141	0.87	0.72–1.05	0.154
13 to 18 months	0.75	0.61–0.92	0.007 **	0.76	0.63–0.93	0.007 **
19 months or more	0.58	0.44–0.75	<0.001 **	0.59	0.46–0.76	<0.001 **
Child’s gender						
Female	Ref.					
Male	0.95	0.79–1.14	0.596			
Child’s race/color						
White	Ref.					
Non-white ^1^	1.17	0.94–1.44	0.152			
Age of the responsible person						
Until 19 years	Ref.					
20 to 29 years	0.83	0.59–1.17	0.290			
30 to 39 years	0.93	0.65–1.31	0.663			
≥40 years	0.78	0.51–1.20	0.255			
Family reference person						
Father	Ref.					
Mother	0.99	0.80–1.21	0.892			
Other	0.95	0.69–1.31	0.757			
Mother’s education						
Low level of education ^2^	Ref.					
Moderate level of education ^3^	1.04	0.81–1.32	0.776			
High level of education ^4^	0.98	0.79–1.22	0.883			
Mother’s current employment status						
Employed	Ref.			Ref.		
Unemployed	1.65	1.13–2.41	0.010 **	1.62	1.10–2.39	0.015 **
Household income (USD)						
Under to 100.00	Ref.					
Between 100.00 and 200.00	0.91	0.73–1.13	0.394			
Between 200.00 and 400.00	0.92	0.73–1.15	0.470			
>400.00	0.62	0.32–1.21	0.162			
Last month’s visit frequency						
<4 visits a month	Ref.					
4 visits (1 a week)	0.91	0.66–1.26	0.567			
PCF attendance time						
Up to 8 months	Ref.					
9 months or more	0.90	0.74–1.08	0.253			
Trust in received orientations						
Do not trust or trust partially	Ref.					
Trust completely and practice them	0.83	0.61–1.13	0.232			
Visit duration						
Less than 60 min	Ref.					
60 min	1.04	0.80–1.33	0.785			
Practice activities proposed by PCF visitor						
No	Ref.					
Yes	0.89	0.64–1.23	0.469			

* Dependent variable of the crude and adjusted models presented; ** Result statistically significant; ^1^ non-white: black, brown, and indigenous; ^2^ low education: illiterate, incomplete/completed primary education, and incomplete secondary education; ^3^ moderate education: completed secondary education or incomplete higher education; ^4^ high education: completed secondary education and incomplete/completed higher education; 95% CI = 95% confidence interval; ref. = reference category; PR = prevalence ratio; PCF = Happy Child Program (Programa Criança Feliz).

**Table 4:** Demographic, socioeconomic, and Happy Child Program adherence characteristics associated with minimum dietary diversity. Federal District, Brazil, 2022.

Explanatory Variables	Minimum Dietary Diversity *					
	Crude Analysis			Multivariate Analysis		
	PR	95% CI	*p*-Value	PR	95% CI	*p*-Value
Child’s age						
Up to 6 months	Ref.					
6 to 12 months	1.90	0.57–6.36	0.295			
13 to 18 months	2.57	0.78–8.45	0.121			
19 months or more	2.45	0.74–8.07	0.141			
Child’s gender						
Female						
Male	0.88	0.72–1.06	0.183			
Child’s race/color						
White	Ref.					
Non-white ^1^	1.27	1.00–1.61	0.050	1.28	1.01–1.62	0.040 **
Age of the responsible person						
Until 19 years	Ref.					
20 to 29 years	1.15	0.70–1.87	0.586			
30 to 39 years	1.04	0.62–1.74	0.873			
≥40 years	1.31	0.08–2.20	0.304			
Family reference person						
Father	Ref.					
Mother	1.24	0.97–1.58	0.086			
Other	1.08	0.74–1.57	0.684			
Mother’s education						
Low level of education ^2^	Ref.					
Moderate level of education ^3^	0.80	0.59–1.08	0.142			
High level of education ^4^	0.95	0.76–1.18	0.653			
Mother’s current employment status						
Employed	Ref.					
Unemployed	1.02	0.77–1.36	0.862			
Household income (USD)						
Under to 100.00	Ref.					
Between 100.00 and 200.00	1.04	0.81–1.33	0.774			
Between 200.00 and 400.00	1.04	0.80–1.34	0.786			
>400.00	0.92	0.55–1.54	0.749			
Last month’s visit frequency						
<4 visits a month	Ref.			Ref.		
4 visits (1 a week)	1.36	1.10–1.67	0.004 **	1.37	1.10–1.71	0.005 **
PCF attendance time						
Up to 8 months	Ref.					
9 months or more	0.91	0.75–1.11	0.357			
Trust in received orientations						
Do not trust or trust partially	Ref.					
Trust completely and practice them	1.45	0.68–3.12	0.335			
Visit duration						
Less than 60 min	Ref.					
60 min	1.08	0.83–1.40	0.567			
Practice activities proposed by PCF visitor						
No	Ref.					
Yes	1.43	0.70–2.96	0.329			

* Dependent variable of the crude and adjusted models presented; ** result statistically significant; ^1^ non-white: black, brown, and indigenous; ^2^ low education: illiterate, incomplete/completed primary education, and incomplete secondary education; ^3^ moderate education: completed secondary education or incomplete higher education; ^4^ high education: completed secondary education and incomplete/completed higher education; 95% CI = 95% confidence interval; ref. = reference category; PR = prevalence ratio; PCF = Happy Child Program (Programa Criança Feliz).

**Table 5:** Demographic, socioeconomic, and adherence characteristics to the Happy Child Program associated with the consumption of ultra-processed foods. Federal District, Brazil, 2022.

Explanatory Variables	Consumption of Ultra-Processed Foods *					
	Crude Analysis			Multivariate Analysis		
	PR	95% CI	*p*-Value	PR	95% CI	*p*-Value
Child’s age						
Up to 6 months	Ref.					
6 to 12 months	1.10	0.64–1.90	0.726			
13 to 18 months	1.18	0.69–2.02	0.534			
19 months or more	1.35	0.79–2.29	0.269			
Child’s gender						
Female	Ref.					
Male	0.78	0.68–0.90	0.001 **	0.79	0.69–0.90	0.001 **
Child’s race/color						
White	Ref.					
Non-white ^1^	1.01	0.88–1.17	0.840			
Age of the responsible person						
Until 19 years	Ref.					
20 to 29 years	0.87	0.68–1.10	0.250			
30 to 39 years	0.88	0.69–1.14	0.333			
≥40 years	1.05	0.82–1.34	0.721			
Family reference person						
Father	Ref.					
Mother	0.97	0.84–1.12	0.679			
Other	0.99	0.79–1.24	0.948			
Mother’s education						
Low level of education ^2^	Ref.					
Moderate level of education ^3^	0.93	0.76–1.13	0.448			
High level of education ^4^	0.99	0.85–1.16	0.936			
Mother’s current employment status						
Employed	Ref.					
Unemployed	0.99	0.83–1.20	0.980			
Household income (USD)						
Under to 100.00	Ref.					
Between 100.00 and 200.00	1.03	0.87–1.22	0.706			
Between 200.00 and 400.00	0.99	0.82–1.18	0.892			
>400.00	1.01	0.73–1.40	0.947			
Last month’s visit frequency						
<4 visits a month	Ref.					
4 visits (1 a week)	0.99	0.79–1.24	0.918			
PCF attendance time						
Up to 8 months	Ref.			Ref.		
9 months or more	1.15	1.01–1.31	0.037 **	1.14	1.00–1.29	0.047 **
Trust in received orientations						
Do not trust or trust partially	Ref.					
Trust completely and practice them	1.31	0.77–2.24	0.310			
Visit duration						
Less than 60 min	Ref.					
60 min	1.03	0.87–1.23	0.700			
Practice activities proposed by PCF visitor						
No	Ref.					
Yes	1.14	0.73–1.79	0.552			

* Dependent variable of the crude and adjusted models presented; ** result statistically significant; ^1^ non-white: black, brown, and indigenous; ^2^ low education: illiterate, incomplete/completed primary education, and incomplete secondary education; ^3^ moderate education: completed secondary education or incomplete higher education; ^4^ high education: completed secondary education and incomplete/completed higher education; 95% CI = 95% confidence interval; ref. = reference category; PR = prevalence ratio; PCF = Happy Child Program (Programa Criança Feliz).

## Data Availability

The original contributions presented in the study are included in the article and Supplementary Material, further inquiries can be directed to the corresponding author.

## Supplementary Materials

The following supporting information can be downloaded at: https://www.mdpi.com/article/10.3390/foods14010138/s1, STROBE checklist.
