# Supplementary material for: Breastfeeding Practices and Food Consumption of Socially Vulnerable Children
Source: Foods. 2025 Jan 6;14(1):138. doi: 10.3390/foods14010138 (PMC11719526; doi:10.3390/foods14010138)
Supplement: Supplementary file 1 [file foods-14-00138-s001.zip › STROBE-checklist.pdf]

STROBE Statement—checklist of items that should be included in reports of observational studies

	Ite m No.	Recommendation	Page No.	Relevant text from manuscript
**Title and abstract**	1	(*a*) Indicate the study's design with a commonly used term in the title or the abstract	page 1	
		(*b*) Provide in the abstract an informative and balanced summary of what was done and what was found	page 1	
**Introduction**				
Background/rationale	2	Explain the scientific background and rationale for the investigation being reported	page 2	
Objectives	3	State specific objectives, including any prespecified hypotheses	page 2	
**Methods**				
Study design	4	Present key elements of study design early in the paper	page 2	This is a cross-sectional observational study
Setting	5	Describe the setting, locations, and relevant dates, including periods of recruitment, exposure, follow-up, and data collection	page 2	
Participants	6	(*a*) *Cohort study*—Give the eligibility criteria, and the sources and methods of selection of participants. Describe methods of follow-up<br>*Case-control study*—Give the eligibility criteria, and the sources and methods of case ascertainment and control selection. Give the rationale for the choice of cases and controls<br>*Cross-sectional study*—Give the eligibility criteria, and the sources and methods of selection of participants	pages 2-3	All families surveyed with children up to 24 months of age were considered eligible for the evaluations. The youngest child was selected in families with more than one child between 0 and 24 months. The inclusion criteria was being eligible for the program throughout the entire research period.
		(*b*) *Cohort study*—For matched studies, give matching criteria and number of exposed and unexposed<br>*Case-control study*—For matched studies, give matching criteria and the number of controls per case	not apply	
Variables	7	Clearly define all outcomes, exposures, predictors, potential confounders, and effect modifiers. Give diagnostic criteria, if applicable	pages 3-4	

Data sources/ measurement	8*	For each variable of interest, give sources of data and details of methods of assessment (measurement). Describe comparability of assessment methods if there is more than one group	pages 3-4	
Bias	9	Describe any efforts to address potential sources of bias	page 4	The data were checked daily to prevent measurement failures or incompleteness, and when necessary, additional data collection was carried out.
Study size	10	Explain how the study size was arrived at	page 3	

Continued on next page

Quantitative variables	11	Explain how quantitative variables were handled in the analyses. If applicable, describe which groupings were chosen and why	page 4	The child's age was stratified to account for dietary diversity in each age group (up to 6 months, 6 to 12 months, 13 to 18 months, 19 months or more). The caregiver's age was also categorized for analysis purposes.
Statistical methods	12	(*a*) Describe all statistical methods, including those used to control for confounding	pages 4 -5	Statistical analyses included descriptive data with prevalence and 95% Confidence Intervals (CI) for categorical variables, and mean with standard deviation (SD) for continuous variables. Poisson regression with robust variance was used to calculate prevalence ratios (PR) for associations between exposure variables (demographic, socioeconomic, and PCF adherence) and outcomes (breastfeeding status, dietary diversity, and UPF consumption).
		(*b*) Describe any methods used to examine subgroups and interactions	page 5	Poisson regression with robust variance
		(c) Explain how missing data were addressed	page 4	Only participants with complete information for the variables included in the model were analyzed.
		(*d*) *Cohort study*—If applicable, explain how loss to follow-up was addressed<br>*Case-control study*—If applicable, explain how matching of cases and controls was addressed<br>*Cross-sectional study*—If applicable, describe analytical methods taking account of sampling strategy	page 5	
		(*e*) Describe any sensitivity analyses	–	
**Results**				

Participants	13*	(a) Report numbers of individuals at each stage of study—eg numbers potentially eligible, examined for eligibility, confirmed eligible, included in the study, completing follow-up, and analysed	page 5	A total of 301 children were assessed.
		(b) Give reasons for non-participation at each stage	not apply	
		(c) Consider use of a flow diagram	not apply	
Descriptive data	14*	(a) Give characteristics of study participants (eg demographic, clinical, social) and information on exposures and potential confounders	page 5	A total of 301 children were assessed, with an average age of 14.54 months. Of these, 51.16% were female, and 69.25% were non-white (Black, Brown, and Indigenous). In more than half of the households (58.99%), the reference person (head of the family) was the mother, with an average age of 29.31 (SD = 8.75) years. Most mothers (86.20%) were unemployed, 27.08% had low education levels, and 83.87% of the households experienced some degree of food insecurity.
		(b) Indicate number of participants with missing data for each variable of interest	not apply	
		(c) *Cohort study*—Summarise follow-up time (eg, average and total amount)	not apply	
Outcome data	15*	*Cohort study*—Report numbers of outcome events or summary measures over time	not apply	
		Case-control study—Report numbers in each exposure category, or summary measures of exposure	not apply	
		Cross-sectional study—Report numbers of outcome events or summary measures	pages 3 -4	Five (current breastfeeding status, exclusive breastfeeding, continued breastfeeding, minimum dietary diversity and consumption of ultra-processed foods).
Main results	16	(*a*) Give unadjusted estimates and, if applicable, confounder-adjusted estimates and their precision (eg, 95% confidence interval). Make clear which confounders were adjusted for and why they were included	pages 7 -11	For the outcomes of current breastfeeding status, minimum dietary diversity, and consumption of ultra-processed foods, both bivariate analysis (first columns) and the final multivariate model are presented.

	(*b*) Report category boundaries when continuous variables were categorized	not apply
	(*c*) If relevant, consider translating estimates of relative risk into absolute risk for a meaningful time period	not apply

Continued on next page

Other analyses	17	Report other analyses done—eg analyses of subgroups and interactions, and sensitivity analyses	not apply	
**Discussion**				
Key results	18	Summarise key results with reference to study objectives	page 11	Most participants had low incomes and lived in situations of food insecurity; nearly all participated in the activities and trusted the guidance provided by the visitors. Our results show that program adherence is associated with greater dietary diversity. However, the same benefit was not observed for BF practices or the consumption of UPF.
Limitations	19	Discuss limitations of the study, taking into account sources of potential bias or imprecision. Discuss both direction and magnitude of any potential bias	page 13	One limitation is that the analysis of food consumption is considered to be only one day, which may not fully reflect the children's eating habits. Another possible limitation is the discomfort in discussing food access or income. To minimize these impacts, efforts were made to clarify the research question and ensure voluntary participation.
Interpretation	20	Give a cautious overall interpretation of results considering objectives, limitations, multiplicity of analyses, results from similar studies, and other relevant evidence	pages 11-13	
Generalisability	21	Discuss the generalisability (external validity) of the study results	page 13	The generalisability of the study results is limited to participants of the Criança Feliz program in the specific region studied, as the sample was drawn exclusively from this group.
**Other information**				
Funding	22	Give the source of funding and the role of the funders for the present study and, if applicable, for the original study on which the present article is based	page 13	This research was supported by Federal District Research Support Foundation (FAP/DF) by grant number 498/2021-FAPDF/SUCTI/COOTEC.

*Give information separately for cases and controls in case-control studies and, if applicable, for exposed and unexposed groups in cohort and cross-sectional studies.

Note: An Explanation and Elaboration article discusses each checklist item and gives methodological background and published examples of transparent reporting. The STROBE checklist is best used in conjunction with this article (freely available on the Web sites of PLoS Medicine at http://www.plosmedicine.org/, Annals of Internal Medicine at http://www.annals.org/, and Epidemiology at http://www.epidem.com/). Information on the STROBE Initiative is available at www.strobe-statement.org.
